# SAE+LSTM: A New Framework for Emotion Recognition From Multi-Channel EEG

**DOI:** 10.3389/fnbot.2019.00037

**Published:** 2019-06-12

**Authors:** Xiaofen Xing, Zhenqi Li, Tianyuan Xu, Lin Shu, Bin Hu, Xiangmin Xu

**Affiliations:** ^1^School of Electronic and Information Engineering, South China University of Technology, Guangzhou, China; ^2^School of Information Science and Engineering, Lanzhou University, Lanzhou, China

**Keywords:** EEG, emotion recognition, neural network, Stack AutoEncoder, LSTM

## Abstract

EEG-based automatic emotion recognition can help brain-inspired robots in improving their interactions with humans. This paper presents a novel framework for emotion recognition using multi-channel electroencephalogram (EEG). The framework consists of a linear EEG mixing model and an emotion timing model. Our proposed framework considerably decomposes the EEG source signals from the collected EEG signals and improves classification accuracy by using the context correlations of the EEG feature sequences. Specially, Stack AutoEncoder (SAE) is used to build and solve the linear EEG mixing model and the emotion timing model is based on the Long Short-Term Memory Recurrent Neural Network (LSTM-RNN). The framework was implemented on the DEAP dataset for an emotion recognition experiment, where the mean accuracy of emotion recognition achieved 81.10% in valence and 74.38% in arousal, and the effectiveness of our framework was verified. Our framework exhibited a better performance in emotion recognition using multi-channel EEG than the compared conventional approaches in the experiments.

## 1. Introduction

Emotion has a great influence on human cognition (Yoo et al., 2014), behavior and communication. Since emotion can reflect information of hobbies, personality, interests and even health, recognition of human emotions can help machines and robots in improving the reliability of human-machine interaction (Yin et al., 2017) and also help them in action processing and social cognition (Urgen et al., 2013). Therefore, research on EEG-based automatic emotion recognition is very important and significance for brain-inspired robots and machines, as it enables them to read people's interactive intentions and states through the wirelessly acquired EEG.

As a subjective feeling, emotion is difficult to be represented by a quantitative model. Researchers often use a two-dimensional space to model emotions (Lang, 1995), where different emotion points can be plotted on a 2D plane consisting of a Valence axis and Arousal axis. Compared with facial expression (Zhang et al., 2016) and speech (Mao et al., 2014), emotion recognition based on physiological signals such as EEG, ECG (electrocardiogram), and EMG (electromyography) (Alzoubi et al., 2012; Chen et al., 2015a; Shu et al., 2018) are more objective and reliable. The main component of the EEG signals are brain rhythm signals from different brain regions, which reflect the activity of the region (Niedermeyer and da Silva, 2005).

The electrical activities of the cortex were propagated through the anatomical structures to the scalp. Therefore, the acquired EEG was a mixture of the source signals from different brain regions, which carried a great deal of redundant information with a low SNR (signal to noise ratio) (Korats et al., 2012). Additionally, the asymmetry features regarding brain regions, such as DASM (differential asymmetry), RASM (rational asymmetry) and DCAU (differential causality) have been explored in the literature on emotion recognition (Zheng et al., 2016; Li et al., 2018), indicating that the spatial information of EEG signals is useful. Decomposing the source signals from different brain regions in the collected EEG could extract useful spatial information while reducing the redundant information in EEG signals, which was considered as one of the key issues in this paper.

On the other hand, the extraction of temporal correlations of spontaneous EEG signals in the context of emotion recognition referred to another key issue. Emotions were affective phenomena varying with time that are caused by a result of stimuli. The context correlation of EEG time sequence reflected the emotion variation. However, most of the commonly used classifiers could only conduct emotion recognition using independent EEG segments, like SVM (support vector machine) or kNN (k-Nearest Neighbor) (Mohammadi et al., 2017). Although there is substantial literature on scalp ERPs, which were highly correlated temporally in the research area of motor control, only a few studies have considered the temporal correlations of spontaneous EEG signals in emotion recognition (Soleymani et al., 2016), and their recognition rate was not adequate. Considering the context correlation of EEG time sequence, making use of the temporal correlation features might provide more effective means in automatic emotion recognition.

In this paper, we present a novel framework for EEG emotion recognition, where SAE is used (Hinton and Salakhutdinov, 2006) to build the linear EEG mixing model and decompose the EEG source signals from the collected EEG signals. Then, followed by the feature extraction, feature sequences of the EEG source signals are obtained. Finally, to explore the temporal correlations in EEG source signal feature sequences, LSTM-RNN (Bengio et al., 2002) is elected as the emotion classifier.

## 2. Related Work

Some recent studies have been working on emotion recognition using EEG signals.

Khosrowabadi et al. presented a biologically inspired feedforward neural network named ERNN to recognize human emotions from EEG. To simulate the short term memory of emotion, a serial-in/parallel-out shift register memory was used in ERNN to accumulate the EEG signals. Compared with other feature extraction methods and feedforward learning algorithms, ERNN achieved the highest accuracy when using the radial basis function (Khosrowabadi et al., 2014).

Soleymani et al. studied how to explore the emotional traces of videos and presented an approach in instantaneously detecting the emotions of video viewers from EEG signals and facial expressions. They utilized LSTM-RNN and continuous conditional random fields (CCRF) to detect emotions automatically and continuously. The results showed that EEG signals and facial expressions carried adequate information for detecting emotions (Soleymani et al., 2016).

Li et al. explored the influence of different frequency bands and number of channels of the EEG signals on emotion recognition. The emotional states were classified into the dimensions of valence and arousal using different combinations of EEG channels. The results showed that the gamma frequency band was preferred and increasing the number of channels could increase the recognition rate (Li et al., 2018).

Independent Component Analysis (ICA) approaches for multi-channel EEG processing are popular, especially for artifact removal and source extraction.

You et al. presented a method of blind signal separation (BSS) for multi-channel EEG, which combined the Wavelet Transform and ICA together. The high-frequency noises were removed from the collected EEG by using the noise filtering function of wavelet transform, so that the ICA could extract the EEG source signals without regard to the problem of noise separation. The experimental results approved the effectiveness of this method in the BBS of multi-channel EEG (You et al., 2004).

Brunner et al. compared three ICA methods (Informax, FastICA and SOBI) with other preprocessing methods (CSP) find out whether and to what extent spatial filtering of EEG data can improve single trial classification accuracy. The results showed that Informax outperformed the other two ICA algorithms (Brunner et al., 2007).

Korats et al. compared the source separation performance of four major ICA algorithms (namely FastICA, AMICA, Extended InfoMax, and JADER) and defined a low bound of data length for robust separation results. AMICA showed an impressive performance with very short data length but required a lot of running time. FastICA took very little time but required twice the data length of AMICA (Korats et al., 2012).

In recent years, autoencoder has drawn more and more attention in biological signal processing, especially in signal reconstruction and feature extraction.

Liu et al. presented a multimodal deep learning approach to construct affective models with the DEAP and SEED datasets to enhance the performance of affective models and reduce the cost of acquiring physiological signals for real-world applications. Using EEG and eye features, the approach achieved mean accuracies of 91.01 and 83.25% on the SEED and DEAP datasets. The experiment results demonstrated that high-level representation features extracted by the BDAE (Bimodal Deep AutoEncoder) network were effective for emotion recognition (Liu et al., 2016).

Majumdar et al. proposed an autoencoder-based framework that simultaneously reconstructed and classified biomedical signals. Using an autoencoder, a new paradigm for signal reconstruction was proposed. It has the advantage of not requiring any assumption regarding the signal as long as there was a sufficient amount of training data. The experiment results showed that the method was better in reconstruction and more than an order of magnitude faster than CS (Compressed Sensing)-based methods. It was capable of providing real-time operations. The method also achieved a satisfactory classification performance (Majumdar et al., 2016).

In these reviewed studies, EEG-based emotional classification has been studied extensively, and corresponding achievements have been realized in the aspects of EEG signal preprocessing, feature extractions, and classifiers. However, decomposition of EEG signals is still a challenge. The current mainly used ICA method assumes the source signals that constitute the mixed EEG signals are independent of each other and do not conform to the normal distribution. But the physiological structure of the brain does not support this hypothesis, as the interconnected cerebral cortex makes the EEG signals have a natural correlation among each other. On the other hand, feature extractions in this area have seldom considered the association and contextual relationships between frames of different EEG signals, which leads to an inadequate utilization of multi-domain information of EEG signals in space-time and the frequency domain. In this work, we tried to explore the method in decomposing EEG signals to source signals and adopt the context correlation of EEG feature sequences to improve emotion recognition.

## 3. Methodology

### 3.1. Framework Design

**Figure 1:**
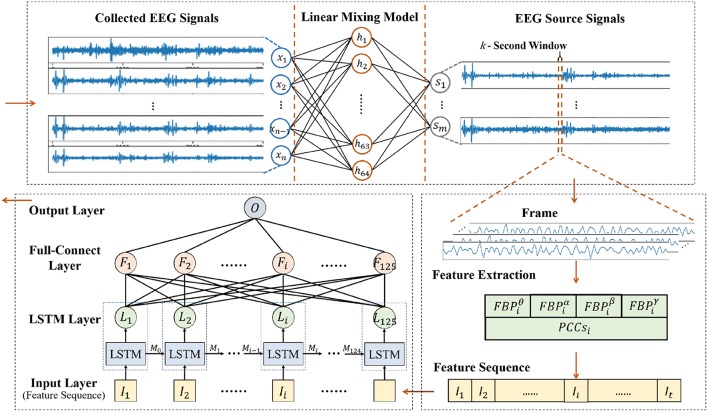
Diagram of our framework.

As shown in ^1^Figure 1, our new framework is made up of three sequential parts, including source signal decomposition, feature extraction and emotion classifier. The details of each part are given below.

In the proposed framework, SAE was used in a linear EEG mixing model to decompose the source signals from the collected EEG signals. LSTM+FC was the main component that was used in the emotion timing model to recognize emotion using the correlation of the EEG feature sequence based on the EEG source signals decomposed by SAE.

### 3.2. Source Signal Decomposition

#### 3.2.1. Linear EEG Mixing Model

**Figure 2:**
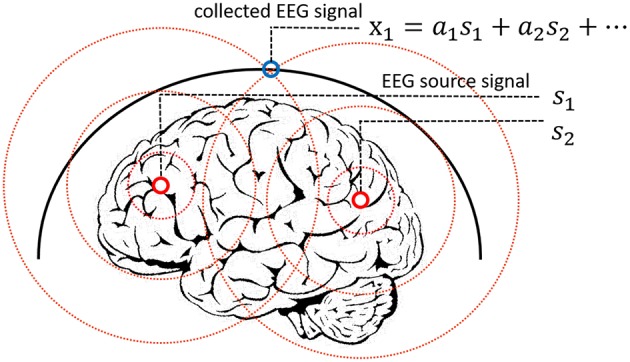
Linear EEG mixing model.

The EEG signal reflects the electrophysiological activity of the cerebral cortex. However, under existing hardware conditions, EEG signals are collected at the scalp instead of the cortex, and there is a skull barrier between the cerebral cortex and the scalp. In fact, the collected EEG signals are the mixture of the EEG source signals. Researchers proposed a linear mixing model to simulate the mixing process, which is widely acknowledged in medical areas (Sanei and Chambers, 2013). In this work, we presented a new method to solve the EEG linear mixing model. The linear EEG mixing model is presented in Figure 2.

The mixture of EEG signals can be written as ^2^(1):

(1) $$\begin{array}{r} \;X=AS \end{array}$$

#### 3.2.2. AutoEncoder

**Figure 3:**
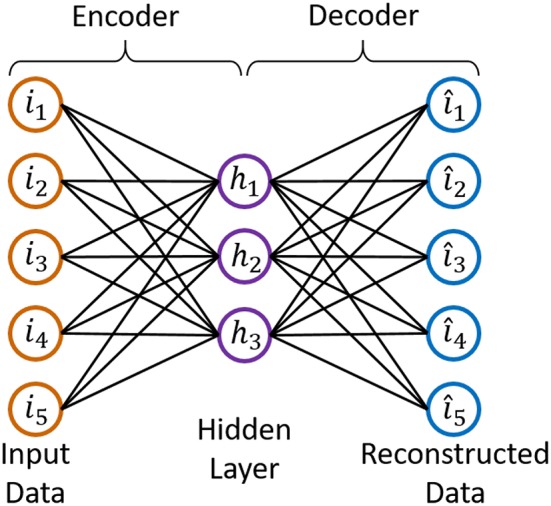
Structure of AutoEncoder.

Autoencoder is an unsupervised neural network consisting of two components, an encoder and a decoder, whose completely symmetrical structure is given in Figure 3. If the reconstructed data is equal to the input data, the output of the “encoder” should be the “code,” which contains all the information about the input data.

When using the linear activation function, the mathematical expression of the encoder is given in ^3^(2) (Ignore the bias).

(2) $$\begin{array}{r} \;H=WI \end{array}$$

From (2), we observe that autoencoder network and linear EEG mixing model have similar expressions. Therefore, we have tried to build and solve the linear EEG mixing model using autoencoder.

#### 3.2.3. Linear EEG Mixing Model Based on Stack AutoEncoder

**Figure 4:**
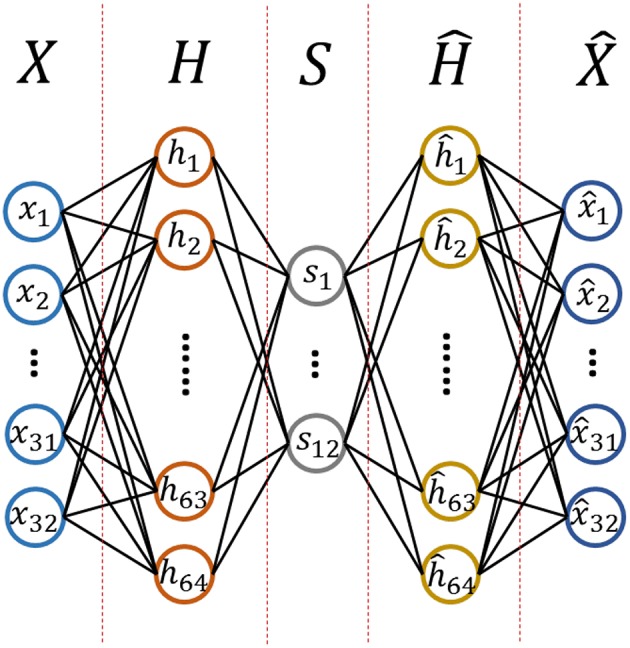
Structure of Stack AutoEncoder.

The purpose of this work is to determine an encoder that allows us to decompose the source signals from the collected EEG signals. To achieve a better performance, an autoencoder is used that consists of multiple layers, called a stacked autoencoder (SAE). The formula of SAE has the same form as the formula of standard autoencoder. The structure and the hyper parameters for the SAE we designed are shown in ^4^Figure 4.

We assumed that the source signals came from 12 different functional brain regions based on previous research (Keil et al., 2002). The 12 regions were formed by crossing hemispheres (left, right) with a horizontal plane (anterior, lateral, posterior) and a vertical plane (inferior, superior) based on recording sites of the international 10–20 system. We made some investigations on the effect of a different number of source channels, such as 6 and 7 source channels. However, the results were not as good as 12 source channels, which was one of the reasons why 12 source channels was selected in our study.

Specifically, *X* is a 32-dimensional vector as a 32-channel EEG signal is used as the input and *S* is a 12-dimensional vector as we discussed before.

#### 3.2.4. Decomposition Results

To conduct the training of our linear EEG mixing model, mini-batch gradient descent was used as the optimizer algorithm, which was an upgraded version of traditional stochastic gradient descent (SGD) and was generally used as the optimizer of the neural network. Mini-batch gradient descent randomly selected a mini batch of data to calculate gradient of the loss function at every step, leading to a fast convergence speed and computational efficiency. As for other optimizers, batch gradient descent needed all data samples to calculate the gradient, which was time-consuming and complicated. Stochastic gradient descent used one sample at each step to reduce the computational complexity and improve the speed, but the drawback was related to its instability and possibility in causing fluctuations. Adam optimizer was faster than SGD and exhibited the advantage of adaptive learning rate, although it might have a convergence problem due to the unstable learning rate (Reddi et al., 2018). In this work, we applied learning rate attenuation in mini-batch gradient descent method to make the model more stable, which turned out to be better than Adam optimizer and other gradient descent methods.

Mean square error (MSE) was used as the loss function. Then, the training data is fed into the model, where the adjusted R-squared between the test data and its reconstructed data is calculated to validate the model. The expression of the adjusted R-square is shown in (3).

(3) $$\begin{array}{r} \;R_{adjusted}^{2}=1-\frac{\left(1-R^{2}\right)\left(N-1\right)}{N-p-1} \end{array}$$

Where *R*^2^ is the sample R-square and *p* is the number of predictors. The value of P was set to 32 in this work since the channel number of EEG signals is 32 in the database of DEAP. *N* is the total sample size. The expression of *R*^2^ is given in (4).

(4) $$R^{2}=1-\frac{\sum^{\text{​}}{\left(X-\hat{X}\right)}^{2}}{\sum^{\text{​}}{\left(X-\;\bar{X}\right)}^{2}}$$

**Figure 5:**
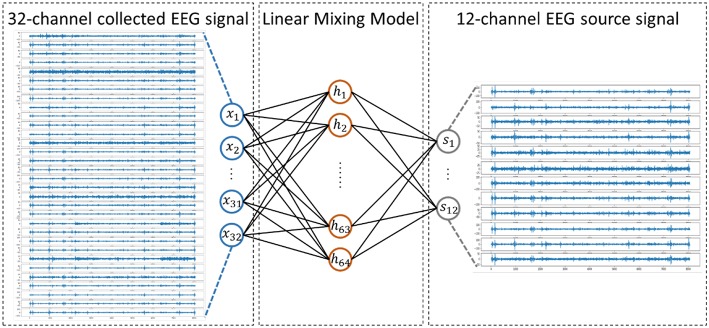
The process of EEG source signal extraction.

When the training data (the 32 channel EEG signals) were fed into the model, the adjusted R-square between the test data and the reconstructed data was calculated to validate the model. Once the adjusted R-square exceeded 0.9, it meant the “code” output by the encoder of our model almost retained all the information of the source EEG signals. In other words, the “code” could represent the EEG source signals successfully and the decomposition was done successfully. The process of EEG source signal extraction is shown in Figure 5.

### 3.3. Feature Extraction

#### 3.3.1. Signal Framing

**Figure 6:**
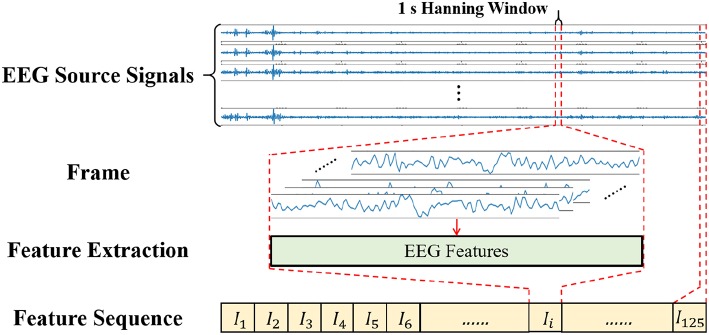
Diagram of Signal Processing.

As a central nervous physiological signal, EEG signal is non-stationary and chaotic. To facilitate signal processing, the EEG signals are always divided into short time frames and it is assumed that the signal within the frame is stationary (Soleymani et al., 2014). Therefore, some signal processing methods for stationary signals are applicable for EEG signal processing. The EEG signal processing steps are shown in Figure 6, where a 1 s window with 50% overlap is applied to the EEG source signals to divide the signals into 125 frames of data. In this work, we also tried sliding data by a 2 s window, 5 s window and so on, while the results turned out no better than the 1s window. The reason might be that the neural network required a larger amount of data, and the 1 s window with 50% overlap could obtain more data than the 2 s window and others. If different experiment settings or models were configured, the choices might be changed flexibly.

After signal framing, the EEG features are extracted from each frame and arranged into a feature sequence. Finally, the feature sequences with 125-frame EEG features are obtained.

#### 3.3.2. Frequency Band Power Feature

**Figure 7:**
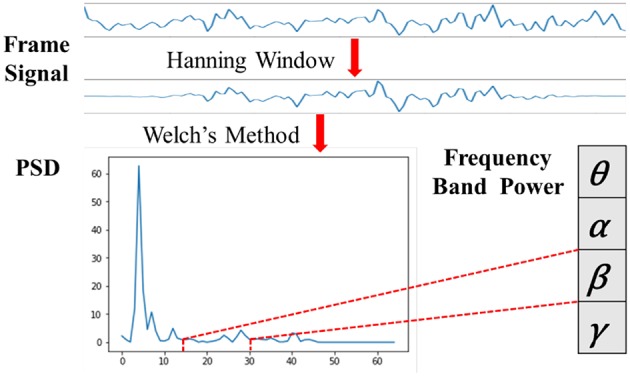
Diagram of the FBP extraction.

Biologically speaking, EEG signals are composed of brain rhythm signals, event related potentials (ERP) and spontaneous electrical activity signals. Many studies (Niedermeyer and da Silva, 2005; Whitten et al., 2011) have proved that changes in brain states are often characterized by rhythmic signals from different brain regions. According to the frequency range from low to high, the EEG signals are divided into five frequency bands of delta waves (δ: 0.5–3.5 Hz), theta waves (θ: 4–7 Hz), alpha waves (α: 8–13 Hz), beta waves (β: 14–30 Hz) and gamma waves (γ: 31–50 Hz). As seen in Figure 7, we applied the Hanning window to each EEG channel and the power spectral density (PSD) was calculated by Welch's method. Then, four frequency band powers (FBP) of the EEG signals were chosen in our experiment.

**Figure 8:**
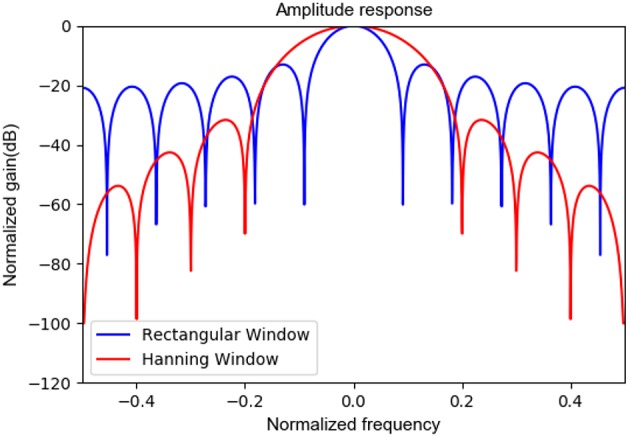
The amplitude response of rectangular window and hanning window.

The Hanning window is most often used in random signals to avoid spectrum leakage. Since EEG signals are typical random signals, the Hanning window was used in this work for data segmentation and band power feature extraction. As the reviewer commented, we plotted the amplitude responses of a rectangular window and a Hanning window for a comparison. In Figure 8, the narrower main window of the rectangular window is more conducive to identifying the specified frequency, however the sidelobe gain is higher and the spectrum leakage is severe, resulting in amplitude information misalignment. The major advantage of the Hanning window is that the spectrum leakage is small, and the main features extracted in this paper are relevant to frequency band energy, so it is appropriate to choose the Hanning window. Of course, other window functions with small spectrum leakage can be also considered.

#### 3.3.3. Channels' Pearson Correlation Coefficient

**Figure 9:**
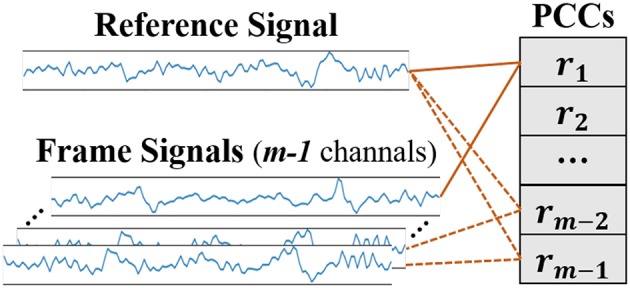
Diagram of the PCCs extraction.

After receiving stimuli, the brain needs to integrate information to understand correctly the emotional significance of the stimuli. According to the ‘binding problem hypothesis' (Singer and Gray, 1995), neurons with similar feature properties will synchronize their discharges under certain specific circumstances, and the functional connectivity of the brain can be estimated using the measure of the synchrony (Gupta et al., 2016). The Pearson correlation coefficient is a measurement on linear correlation between two signals and can be used to measure the inter-channel EEG correlations (Bonita et al., 2014; Chen M. et al., 2015). As seen in Figure 9, one of the frame signals is selected as the reference signal and the Pearson correlation coefficients (PCC) between signals can be calculated by (5).

(5) $$\begin{array}{r} \;PCC=\frac{\sum_{i=1}^{N}\left(x_{i}-\bar{x}\right)\left(y_{i}-ȳ\right)}{\sqrt{\sum_{i=1}^{N}{\left(x_{i}-\bar{x}\right)}^{2}}\sqrt{\sum_{i=1}^{N}{\left(y_{i}-ȳ\right)}^{2}}} \end{array}$$

### 3.4. Classifier

#### 3.4.1. Emotion Timing Model

In emotional situations, the hippocampal complex and amygdala interact in subtle but important ways. Specifically, the hippocampal complex can influence the amygdala's response when emotional stimuli are encountered (Phelps, 2004). Therefore, we assume that the present emotional status is influenced by the previous emotional status, and EEG under previous emotion status might have correlations with those under present emotion state as EEG could reflect the emotion status while EEG context information could also be adopted in emotion recognition (Li et al., 2017b), so the EEG feature sequence was viewed as containing information on emotion changes in this paper. Based on this assumption, we then propose an emotion timing model. To simulate our emotion timing model, a classifier is needed which can take full advantage of the context correlations in EEG feature sequences.

#### 3.4.2. Long Short-Term Memory Network

**Figure 10:**
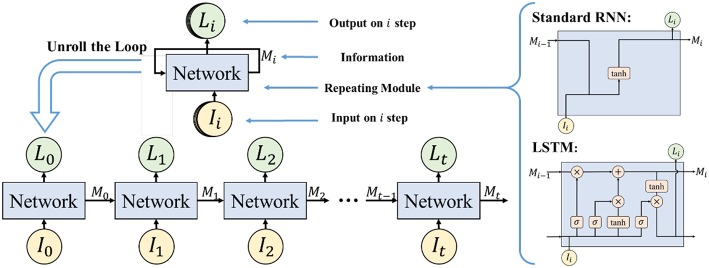
The structure of regular RNN and LSTM. Reproduced with permission (Li et al., 2018) Copyright 2018, Springer.

The Long Short-Term Memory network (LSTM) is applied to do the emotion classification, which is an improvement on the Recurrent Neural Network (RNN). RNN has the problem of long-term dependencies (Bengio et al., 2002) so it is not suitable for time series analysis, while LSTM can solve the problem due to the design of its repeating module. LSTM is thus adopted in our work to calculate the context correlations of EEG feature sequence. The structure of a regular RNN and our LSTM model in this study is shown in Figure 10.

#### 3.4.3. Emotion Classifier Based on LSTM

To recognize emotion using the correlation of the EEG feature sequence, a deep neural network for emotion recognition based on LSTM is proposed. The first layer of the deep neural network is the LSTM layer, which is used to mine the context correlation in the input EEG feature sequence. The second layer is the full-connect layer, which is used to integrate information and act as the major role of the classifier.

**Figure 11:**
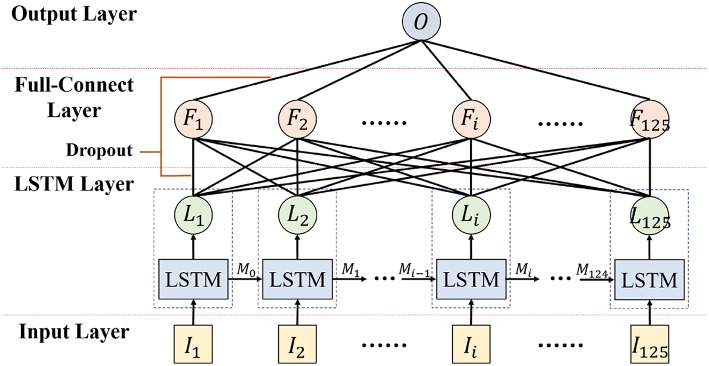
Emotion Classifier based on LSTM.

The detailed hyper parameter settings for our neural network model are illustrated in Figure 11. In the LSTM layer, 125 LSTM cells are set to correspond to 125 frame features in each sequence. In the full-connect layer, connection units are set with the same number. Finally, the sigmoid activation function is used in the output layer. For classifier training, the mini-batch gradient descent optimizer and the MSE loss function have been also used.

“Dropout” was added in the LSTM and full-connect layers to avoid over-fitting. The training epochs of LSTM were set to a few thousand. In the first few hundred epochs, a high learning rate was set to speed up the training procedure, and then it was slowly changed to a lower rate to achieve more robust results. When the training AUC met the set goal, the training was completed.

### 3.5. Model Training

#### 3.5.1. Hyper-Parameter Tuning

The SAE and LSTM models were trained separately, and the parameters were set or tuned according to certain rules or bases to ensure their optimization.

1. The SAE model was an unsupervised model trained via the back propagation of the reconstruction error. The hyper-parameter setting is described below: the input layer contained 32 units determined by the number of EEG data channels in the DEAP dataset. There were 64 units in the first hidden layer which were tuned by the reconstruction error. The second hidden layer had 12 units, which was consistent with the 12 functional brain zones. To conduct the training of our linear EEG mixing model, the mini-batch gradient descent was used as the optimizer algorithm and the mean square error (MSE) was applied as the loss function.
2. The LSTM model was a supervised model. Its time step was set to 125, as 125 data segments were achieved under the conditions whereby each EEG data in DEAP had a length of 63 s, and a 1 s time window with 0.5 s step size was adopted. The hidden layer of LSTM had 125 units, which was tuned by the reconstruction error.

#### 3.5.2. Over-Fitting Handling

The proposed framework could effectively solve the over-fitting issue. The SAE model was trained using the reconstruction error, and the sparse and penalty constraints were added to avoid the over-fitting problem. In the training of the LSTM+FC model, three aspects of work had been conducted to handle the over-fitting/over-training issues: (1) A 1 s window with 50% overlap was applied to the EEG data segmentation, which augmented the size of data samples and guaranteed the amount of data used in model training. (2) “Dropout” operations were added in the training of the LSTM and full-connect layers to avoid overfitting, which can be seen in Figure 11. (3) Regularization items of the parameters had been added. (4) 10-fold cross validation was used to verify our approach, and the result of cross validation could be considered that these results were highly probable without over-fitting.

#### 3.5.3. Training Visualization

**Figure 12:**
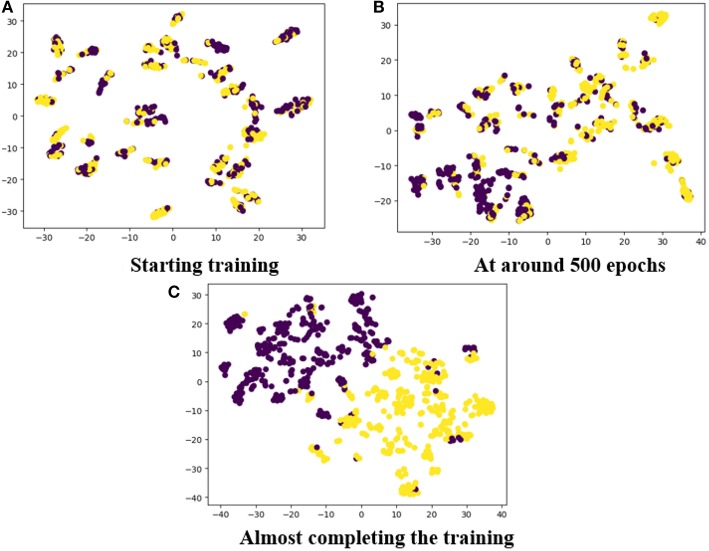
The feature clustering during training: **(A)** Starting training; **(B)** at around 500 epochs; **(C)** almost completing the training.

To show how our proposed method handled the data during network training, the training procedure was visualized by plotting the feature clustering in different epochs. In Figure 12, the features in connections with positive and negative emotions have been clustered into two categories which are represented by two colors. It can be observed that after a few thousand epochs, the features were clearly classified by our model.

## 4. Experimental and Results

The effectiveness of our framework was evaluated on the DEAP dataset. At first, we compared our framework with other methods on a trial-oriented emotion recognition task. Then three experiment settings were designed to verify the validity of LSTM and SAE in an emotion recognition task using different EEG features.

### 4.1. Experimental Dataset

We used the EEG data from the DEAP dataset to validate our framework (Koelstra et al., 2012). DEAP is a database using different kinds of physiological signals for human affective state analysis. It contains 32-channel electroencephalogram (EEG) and 8-channel peripheral physiological signals of 32 subjects. Each subject was required to watch 40 one-minute excerpts of music videos during which their signals were recorded. Subjects rated each video in terms of valence, arousal, dominance, liking with the rating distributed from 1 to 9 in each dimension.

The EEG signals in the DEAP database were downsampled to 128 Hz, and a 4.0–45 Hz band-pass filter was applied. The data were then segmented into several 63 s trials, where the 3 s pre-trials were removed and the following 60 s trials were kept for further processing. Since EEG signals might be contaminated by other signals such as EOG (Li et al., 2017a; Samuel et al., 2017), the EOG noise was eliminated by ICA in the DEAP dataset to ensure that EEG data can better represent the emotions of the subjects.

**Figure 13:**
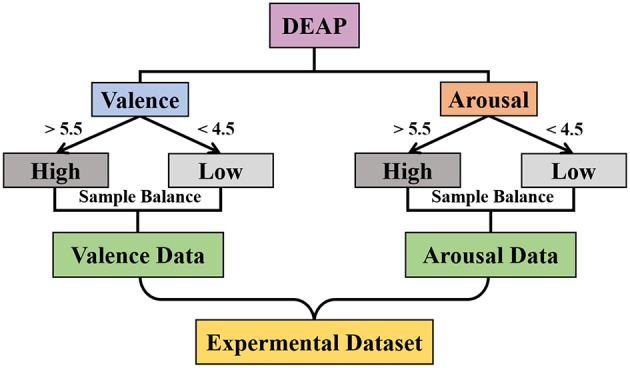
Diagram of the experimental dataset selection.

As shown in Figure 13, the experimental datasets were selected from DEAP, where we divided the trials into two classes based on the value of valence (or arousal) and labeled “High” if the valence (or arousal) value was higher than 5.5 and “Low” if it was lower than 4.5. Then, the down-sampling method was used to balance the number of samples of both “High" and “Low” and we obtained the valence (or arousal) dataset.

### 4.2. Emotion Recognition Results

We selected some relevant studies which had similar experimental settings for a comparison. We used the 10-fold cross validation method to validate results in our classification. The 10-fold cross-validation method applied in our work was the regular cross-validation method which was normally adopted in relevant studies (Li et al., 2017b, 2018). Specifically, the data samples contained with all subjects' information were randomly split by 10-fold cross-validation method into 10 folds, where 9 folds were for training and 1 for testing. The validation process was repeated ten times to achieve an average result. The results were calculated by ^5^(6).

(6) $$\begin{array}{r} \;AUC_{mean}=\frac{1}{10}\overset{10}{\underset{k=1}{\sum }}\frac{N_{correct}^{k}}{N_{test}} \end{array}$$

**Figure 14:**
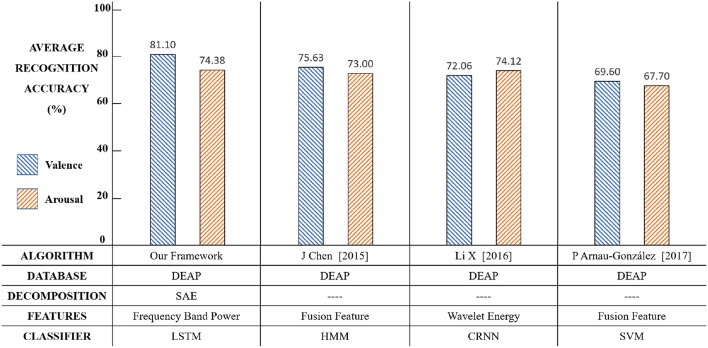
The results comparison among relevant methods.

The average accuracy results of our new framework with a comparison of other conventional methods are shown in Figure 14. The results show that our framework exhibits an effective performance.

Compared with relevant methods, our framework achieves the best performance in emotion recognition using both valence (81.10%) and arousal (74.38%). The reason might be as follows. Chen et al. used HMM to build the relationship between the present and previous emotion states (Chen et al., 2015b). However, each step output of HMM was only related to some of the previous states, thus the classifier could not automatically learn like LSTM. Li et al. proposed a CRNN framework for emotion recognition (Li et al., 2017b), but CNN required a large quantity of training data to extract features and the DEAP dataset cannot satisfy that. P Arnau-Gonzlez (2017) studied the method of EEG feature fusion and achieved the best accuracy using SVM (Arnau-Gonzlez et al., 2017), but the SVM classifier was not able to explore the context correlations of the EEG feature sequence, therefore its performance was limited.

The main purpose of setting up this framework was for valence classification. Theoretically, the effect of EEG spatial information on valence classification was more obvious. The results in Figure 14 showed that our classification accuracy in valence (81.10%) was better than relevant studies. Meanwhile, the framework did not affect and even slightly improved the arousal classification performance. The innovative point was that this framework effectively utilized the time domain and space domain information of EEG signals by a linear EEG mixing model based on SAE and an emotion timing model based on LSTM, which significantly improved the valence classification and did not affect, or even slightly improved, the arousal classification.

### 4.3. Verification Experiment

In order to further verify the effectiveness of our framework, we designed three sets of experiments and made a comparison of the performance on different emotional dimensions by statistical analysis.

#### 4.3.1. First Experiment - SVM and LSTM Classifier

**Figure 15:**
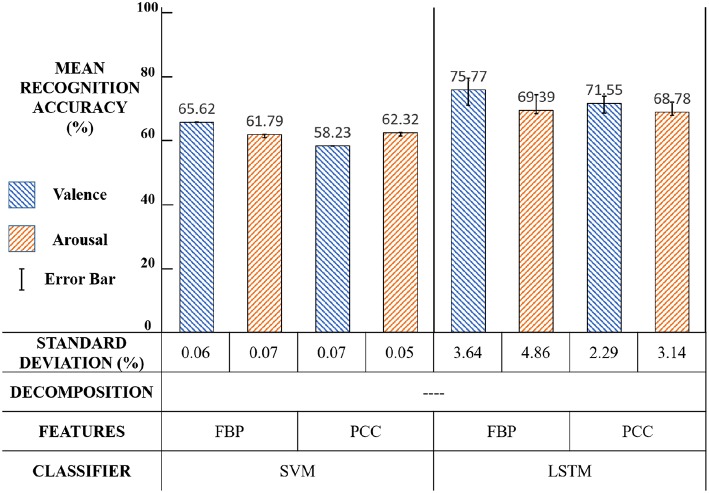
First experiment: settings and results.

The first experiment was designed to demonstrate the validity of our LSTM classifier. The experiment settings and results are shown in Figure 15 and the significance test results are shown in Table 1.

**Table 1:**
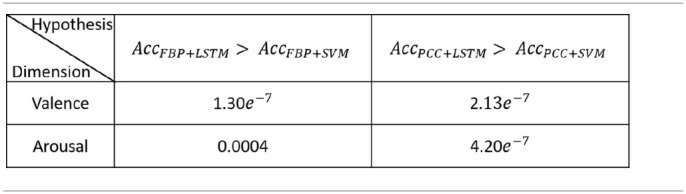
First experiment: significance test results.

The values in Table 1 are p-values. The p-value in different experiment setups was the probability of paired sample *t*-tests for different experiment setups, which was calculated by results of the average and standard deviation of the 10-fold cross validation. The main idea of the *t*-test was to state recognition results under two conditions to get the approximate distribution of each condition and to calculate the probability that two distributions have significant difference. When *p* < 0.01 (or 0.05), it can generally be concluded the emotion recognition rate of our method was significantly higher than other methods by using different EEG features in both valence and arousal.

Compared with SVM, the emotion recognition accuracy of LSTM was significantly (*p* < 0.01) higher in both valence and arousal, which proves that exploring the correlations in the EEG feature sequence was more effective than merely integrating the recognition result of each EEG feature frame.

Using LSTM can model emotion in the time dimension and extract the emotion feature of each time step, so that our classifier can integrate the entire feature sequence information. This result agrees with our previous assumption that the change of emotion is continuous.

#### 4.3.2. Second Experiment - ICA and SAE

The second experiment was designed to evaluate the performance of our SAE based model for EEG source signal decomposition, which contained two parts.

**Figure 16:**
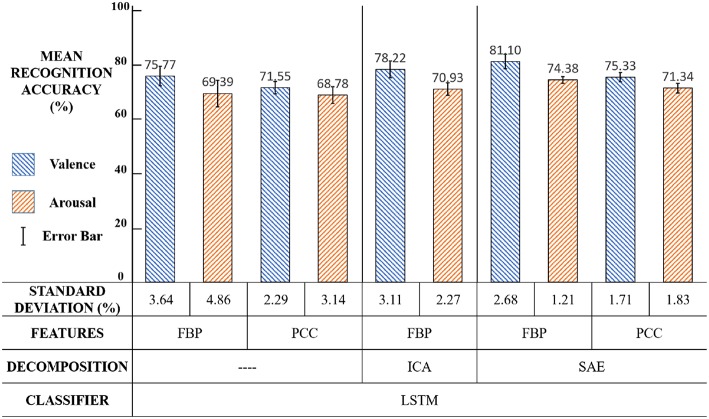
Second experiment: settings and results.

In the first part, we compared the classification performances among methods with EEG decomposition via SAE or ICA or without EEG decomposition. The results in Figure 16 showed that the SAE based EEG source signal decomposition method achieved better performance than the ICA methods or non-decomposing methods, especially in the case of using FBP features. The statistically significance test results in Table 2 further verified the results (*p* < 0.01).

**Table 2:**
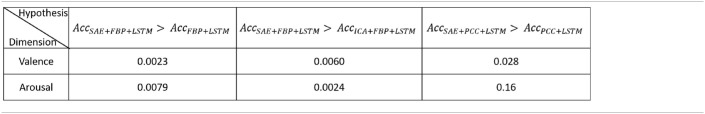
Second experiment: significance test results.

Using SAE for EEG source signal decomposition, in fact, was to encode the EEG channel. The spatial characteristics of the EEG signal, in other words, EEG channel correlations, were also extracted at this time, which was the reason why using EEG source signals could improve the emotion recognition accuracy.

EEG source signal decomposition was an important step in our framework, which took extra time cost. Luckily, using SAE for EEG source signal decomposition would reduce the number of channels of EEG signals that need to be processed, and saved time for feature extraction.

**Figure 17:**
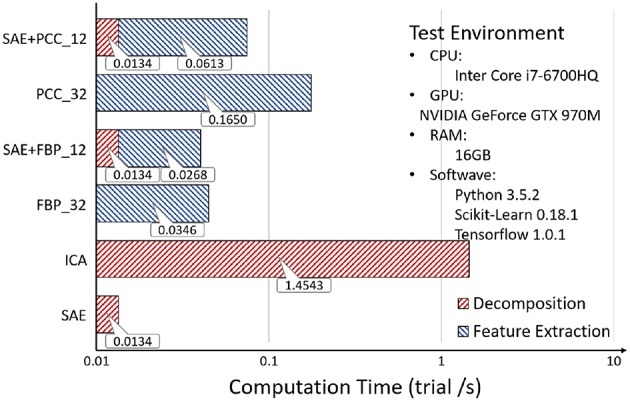
Second experiment: Computation Time.

In the second part, we counted the total computation time of EEG source signal decomposition and feature extractions, as can be seen in Figure 17, where it was observed that although the decomposition process costs extra time, it reduces the time spent in feature extraction, especially for complex features. The operation speed of SAE is two orders of magnitude faster than that of ICA. The result is explained as follows:^6^

According to the experimental results, using SAE for EEG source signal decomposition could improve the emotion recognition accuracy while ensuring fast recognition speed.

The number of parameters in both the SAE and LSTM models were recorded, where the values were 2040376 and 5804 for LSTM and SAE, respectively. We tried to estimate the parameter number of other models in the relevant literature. However, many of them did not provide the whole parameter settings, especially the parameters regarding hidden layers, so the numbers could not be calculated, and hence a comprehensive comparison on the computational complexity using the number of parameters has not been achieved.

#### 4.3.3. Third Experiment - FBP and PCC

**Figure 18:**
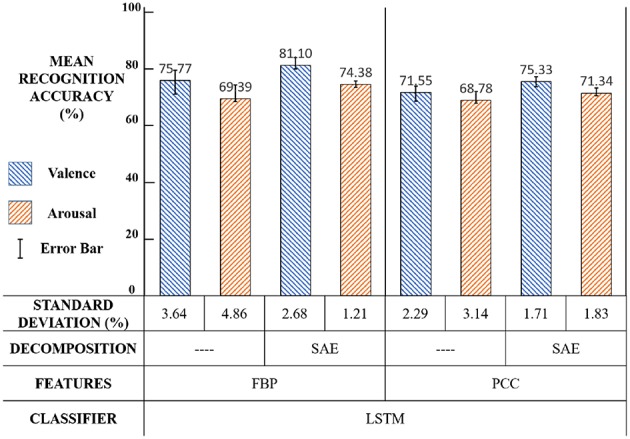
Third experiment: Settings and results.

The third experiment was designed to compare the performance of FBP and PCC in our new framework. The experiment settings and results are illustrated in Figure 18 and the significance test results are shown in Table 3.

**Table 3:**
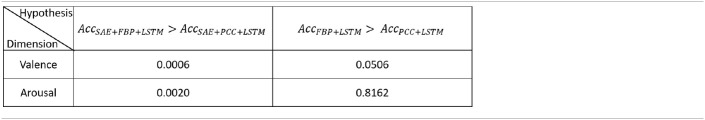
Third experiment: significance test results.

We can see that in Figure 16 and Table 3, compared with the PCC feature, the FBP feature performs better (*p* < 0.01). The reason may be that FBP is a frequency-domain feature while PCC is a spatial-domain feature. Combining EEG source signal with FBP, “SAE+FBP" can reflect the features of EEG in the spatial-frequency domain, like DASM feature and RASM feature (Lin et al., 2010). While “SAE+PCC" can only reflect EEG in the spatial domain. Therefore, we view the frequency-domain features more suitable for our framework.

## 5. Discussion

In this work, we obtained EEG-based emotion recognition rates of 81.10% in valence and 74.38% in arousal. The current recognition rates of EEG-based emotion recognition methods are still not adequate for real applications. One of the major problems is related to individual differences, which can be minimized via experiment paradigm design or calibrations that can remove the effects of EEG baseline variations on different subjects. On the other hand, the emotion classification accuracies of these methods are difficult to evaluate in an objective way, since there are no universal standard test datasets in the area, and the evaluation steps of the related work in the literature are different. Eliminating individual differences and establishing standard test sets represent important future work for EEG-based emotion recognition.

Compared with valence, our framework does not exhibit high recognition accuracy in arousal. On the one hand, the EEG features we used might not be enough. Trying more complex features, such as the EEG spectral asymmetry index (SASI) (Orgo et al., 2015), the derived features of bispectrum (Kumar et al., 2016) and the wavelet entropy features (Hosseini and Naghibi-Sistani, 2011), may be more effective. On the other hand, our classifier network may be not complex enough. Using Bidirectional recurrent neural networks (Schuster and Paliwal, 1997), like Bidirectional LSTM (Sak et al., 2014), as classifier may achieve better recognition performance. All of our experiments were conducted on the DEAP dataset. In order to evaluate our framework more systematically and comprehensively, an additional EEG dataset with emotional tags is needed.

In this work, we focused on valence and arousal based on the literature (Chen et al., 2015b; Li et al., 2017b; Mohammadi et al., 2017). Since most relevant studies made the same choice, it is fairer to compare their results with ours in the valence and arousal dimensions. Of course, dominance and other dimensions would be considered in the future work.

## 6. Conclusions

In this paper, we present a novel emotion recognition framework consisting of a linear EEG mixing model and an emotion timing model. The SAE-based linear EEG mixing model can be used for decomposition of EEG source signals and extracting EEG channel correlations, and it can also improve computation efficiency in feature extraction and upgrade the emotion recognition performance. The emotion timing model is simulated by LSTM, which increased the recognition accuracy by exploring the context correlations of the EEG feature sequence. The comparison results in our experiment approved the effectiveness of our framework, especially in the valence recognition task. This work can promote the development of brain-inspired robots, especially in human-robot interaction.

## Author Contributions

XfX, ZL, and LS designed the framework, conducted experiments and wrote the manuscript; ZL and TX carried out experiments; XfX, BH, and XmX analyzed the results and presented the discussion and conclusion parts.

### Conflict of Interest Statement

The authors declare that the research was conducted in the absence of any commercial or financial relationships that could be construed as a potential conflict of interest.

## Footnotes

^1^*x*_1_~*x*_*n*_ represent the *n*th-channel EEG signals, *s*_1_~*s*_*m*_ represent the *m*th-channel EEG source signals, $FBP_{i}^{\theta }$, $FBP_{i}^{\alpha }$, $FBP_{\beta }^{2}$ and $FBP_{i}^{\gamma }$ represent the frequency band power of theta, alpha, beta and gamma band in the *i*th frame, respectively, *PCCs*_*i*_ represent the Pearson correlation coefficient of each channel in the *i*th frame, *I*_1_~*I*_*t*_ represent each frame of the feature sequence, *L*_1_~*L*_*t*_ represent each step of the unrolled LSTM layer, *F*_1_~*F*_*t*_ represent each unit of the Full-Connect layer, *O* represents the output layer of the classifier.

^2^*X* refers to the collected EEG signals, *A* refers to the mixing matrix and *S* denotes the EEG source signals.

^3^*I* is the input data, *H* is the output of the hidden layer, *W* is the transformation matrix of the encoder.

^4^*X* refers to the collected EEG signals, *H* refers to the output signals of the first hidden layer, *S* refers to the EEG source signals, $\hat{X}$ and Ĥ refers to the reconstructed data of *X* and *H*.

^5^*AUC*_*mean*_ represents the average recognition accuracy, *N*_*test*_ represents the number of the testing samples, $N_{correct}^{k}$ is the number of samples that have been classified correctly.

^6^*FBP*_32 represents the FBP feature extraction of 32-channel collect EEG signals, *PCC*_12 represents the PCC feature extraction of 12-channel EEG source signals, *SAE* represents the EEG source signal decomposition using SAE based approach and *ICA* represents the EEG source signal decomposition using an ICA-based approach. When using ICA to decompose EEG source signals, the EEG data needed to be whitened. Since there existed significant variations in EEG amplitudes among different subjects or among different trials under the same subject, it was necessary to resolve the ICA model when decomposing different EEG data records. However, the SAE method required only one solution, therefore its time consumption was much lower than ICA.

## References

[B1] Alzoubi O. D'Mello S. K. Calvo R. A. (2012). Detecting naturalistic expressions of nonbasic affect using physiological signals. IEEE Trans. Affect. Comput. 3, 298–310. 10.1109/T-AFFC.2012.4

[B2] Arnau-Gonzlez P. Arevalillo-Herrez M. Ramzan N. (2017). Fusing highly dimensional energy and connectivity features to identify affective states from eeg signals. Neurocomputing 244, 81–89. 10.1016/j.neucom.2017.03.027

[B3] Bengio Y. Simard P. Frasconi P. (2002). Learning long-term dependencies with gradient descent is difficult. IEEE Trans. Neural Netw. 5, 157–166. 10.1109/72.279181 18267787

[B4] Bonita J. D. Ambolode L. C. C. II. Rosenberg B. M. Cellucci C. J. Watanabe T. A. A. Rapp P. E.. (2014). Time domain measures of inter-channel eeg correlations: a comparison of linear, nonparametric and nonlinear measures. Cogn. Neurodyn. 8, 1–15. 10.1007/s11571-013-9267-8 24465281 PMC3890093

[B5] Brunner C. Naeem M. Leeb R. Graimann B. Pfurtscheller G. (2007). Spatial filtering and selection of optimized components in four class motor imagery eeg data using independent components analysis. Patt. Recogn. Lett. 28, 957–964. 10.1016/j.patrec.2007.01.002

[B6] Chen J. Hu B. Moore P. Zhang X. Ma X. (2015a). Electroencephalogram-based emotion assessment system using ontology and data mining techniques. Appl. Soft Comput. 30, 663–674. 10.1016/j.asoc.2015.01.007

[B7] Chen J. Hu B. Xu L. Moore P. Su Y. (2015b). “Feature-level fusion of multimodal physiological signals for emotion recognition,” in IEEE International Conference on Bioinformatics and Biomedicine (Washington DC), 395–399.

[B8] Chen M. Han J. Guo L. Wang J. Patras I. (2015). “Identifying valence and arousal levels via connectivity between eeg channels,” in International Conference on Affective Computing and Intelligent Interaction (Xi'an), 63–69.

[B9] Gupta R. Laghari K. U. R. Falk T. H. (2016). Relevance vector classifier decision fusion and eeg graph-theoretic features for automatic affective state characterization. Neurocomputing 174, 875–884. 10.1016/j.neucom.2015.09.085

[B10] Hinton G. E. Salakhutdinov R. R. (2006). Reducing the dimensionality of data with neural networks. Science 313, 504–507. 10.1126/science.1127647 16873662

[B11] Hosseini S. A. Naghibi-Sistani M. B. (2011). Emotion recognition method using entropy analysis of eeg signals. Int. J. Image Graph. Signal Process. 3:30 10.5815/ijigsp.2011.05.05

[B12] Keil A. Bradley M. M. Hauk O. Rockstroh B. Elbert T. Lang P. J. (2002). Large-scale neural correlates of affective picture processing. Psychophysiology 39, 641–649. 10.1111/1469-8986.3950641 12236331

[B13] Khosrowabadi R. Chai Q. Kai K. A. Wahab A. (2014). Ernn: a biologically inspired feedforward neural network to discriminate emotion from eeg signal. IEEE Trans. Neural Netw. Learn. Syst. 25, 609–620. 10.1109/TNNLS.2013.2280271 24807454

[B14] Koelstra S. Muhl C. Soleymani M. Lee J. S. Yazdani A. Ebrahimi T. (2012). Deap: a database for emotion analysis ;using physiological signals. IEEE Trans. Affect. Comput. 3, 18–31. 10.1109/T-AFFC.2011.15

[B15] Korats G. Le Cam S. Ranta R. Hamid M. (2012). “Applying ica in eeg: choice of the window length and of the decorrelation method,” in International Joint Conference on Biomedical Engineering Systems and Technologies (Vilamoura: Springer), 269–286.

[B16] Kumar N. Khaund K. Hazarika S. M. (2016). Bispectral analysis of eeg for emotion recognition. Proced. Comput. Sci. 84, 31–35. 10.1016/j.procs.2016.04.062

[B17] Lang P. J. (1995). The emotion probe. Studies of motivation and attention. Am. Psychol. 50, 372–385. 7762889 10.1037//0003-066x.50.5.372

[B18] Li M. Xu H. Liu X. Lu S. (2018). Emotion recognition from multichannel eeg signals using k-nearest neighbor classification. Tech. Health Care 26(Suppl. 1):509–519. 10.3233/THC-174836 29758974 PMC6027901

[B19] Li X. Samuel O. W. Zhang X. Wang H. Fang P. Li G. (2017a). A motion-classification strategy based on semg-eeg signal combination for upper-limb amputees. J. Neuroeng. Rehabilit. 14:2. 10.1186/s12984-016-0212-z 28061779 PMC5219671

[B20] Li X. Song D. Zhang P. Yu G. Hou Y. Hu B. (2017b). “Emotion recognition from multi-channel eeg data through convolutional recurrent neural network,” in IEEE International Conference on Bioinformatics and Biomedicine (Shenzhen), 352–359.

[B21] Li Z. Tian X. Shu L. Xu X. Hu B. (2018). “Emotion Recognition from EEG Using RASM and LSTM,” in Internet Multimedia Computing and Service. ICIMCS 2017. Communications in Computer and Information Science, Vol. 819, eds Huet B. Nie L. Hong R. (Singapore: Springer).

[B22] Lin Y. P. Wang C. H. Jung T. P. Wu T. L. Jeng S. K. Duann J. R.. (2010). Eeg-based emotion recognition in music listening. IEEE Trans. BioMed. Eng. 57:1798. 10.1109/TBME.2010.2048568 20442037

[B23] Liu W. Zheng W.-L. Lu B.-L. (2016). Emotion recognition using multimodal deep learning. in International Conference on Neural Information Processing (Kyoto: Springer), 521–529.

[B24] Majumdar A. Gogna A. Ward R. (2016). Semi-supervised stacked label consistent autoencoder for reconstruction and analysis of biomedical signals. IEEE Trans. Biomed. Eng. 99, 1–1. 10.1109/TBME.2016.2631620 27893378

[B25] Mao Q. Dong M. Huang Z. Zhan Y. (2014). Learning salient features for speech emotion recognition using convolutional neural networks. IEEE Transact. Multi. 16, 2203–2213. 10.1109/TMM.2014.2360798

[B26] Mohammadi Z. Frounchi J. Amiri M. (2017). Wavelet-based emotion recognition system using eeg signal. Neural Comput. Appl. 28, 1985–1990. 10.1007/s00521-015-2149-8

[B27] Niedermeyer E. da Silva F. L. (2005). Electroencephalography: Basic Principles, Clinical Applications, and Related Fields. Lippincott Williams & Wilkins.

[B28] Orgo L. Bachmann M. Lass J. Hinrikus H. (2015). “Effect of negative and positive emotions on eeg spectral asymmetry,” in 2015 37th Annual International Conference of the IEEE Engineering in Medicine and Biology Society (EMBC) (Milan: IEEE), 8107–8110. 10.1109/EMBC.2015.7320275 26738175

[B29] Phelps E. A. (2004). Human emotion and memory: interactions of the amygdala and hippocampal complex. Curr. Opin. Neurobiol. 14, 198–202. 10.1016/j.conb.2004.03.015 15082325

[B30] Reddi S. J. Kale S. Kumar S. (2018). “On the convergence of adam and beyond,” in International Conference on Learning Representations (Vancouver, BC).

[B31] Sak H. Senior A. Beaufays F. (2014). “Long short-term memory recurrent neural network architectures for large scale acoustic modeling,” in Fifteenth Annual Conference of the International Speech Communication Association (Singapore).

[B32] Samuel O. W. Geng Y. Li X. Li G. (2017). Towards efficient decoding of multiple classes of motor imagery limb movements based on eeg spectral and time domain descriptors. J. Med. Syst. 41:194. 10.1007/s10916-017-0843-z 29080913

[B33] Sanei S. Chambers J. A. (2013). EEG Signal Processing. John Wiley & Sons.

[B34] Schuster M. Paliwal K. K. (1997). Bidirectional recurrent neural networks. IEEE Trans. Signal Process. 45, 2673–2681.

[B35] Shu L. Xie J. Yang M. Li Z. Li Z. Liao D.. (2018). A review of emotion recognition using physiological signals. Sensors 18:2074. 10.3390/s18072074 29958457 PMC6069143

[B36] Singer W. Gray C. M. (1995). Visual feature integration and the temporal correlation hypothesis. Ann. Rev. Neurosci. 18:555. 7605074 10.1146/annurev.ne.18.030195.003011

[B37] Soleymani M. Asghari-Esfeden S. Fu Y. Pantic M. (2016). Analysis of eeg signals and facial expressions for continuous emotion detection. IEEE Trans. Affect. Comput. 7, 17–28. 10.1109/TAFFC.2015.2436926

[B38] Soleymani M. Asghariesfeden S. Pantic M. Fu Y. (2014). “Continuous emotion detection using eeg signals and facial expressions,” in IEEE International Conference on Multimedia and Expo (Chengdu), 1–6.

[B39] Urgen B. Plank M. Ishiguro H. Poizner H. Saygin A. (2013). Eeg theta and mu oscillations during perception of human and robot actions. Front. Neurorobot. 7:19. 10.3389/fnbot.2013.00019 24348375 PMC3826547

[B40] Whitten T. A. Hughes A. M. Dickson C. T. Caplan J. B. (2011). A better oscillation detection method robustly extracts eeg rhythms across brain state changes: the human alpha rhythm as a test case. Neuroimage 54:860. 10.1016/j.neuroimage.2010.08.064 20807577

[B41] Yin Z. Wang Y. Liu L. Zhang W. Zhang J. (2017). Cross-subject eeg feature selection for emotion recognition using transfer recursive feature elimination. Front. Neurorobot. 11:19. 10.3389/fnbot.2017.00019 28443015 PMC5385370

[B42] Yoo J. Kwon J. Choe Y. (2014). Predictable internal brain dynamics in eeg and its relation to conscious states. Front. Neurorobot. 8:18. 10.3389/fnbot.2014.00018 24917813 PMC4043151

[B43] You R. Xu S. Chen Z. (2004). Blind signal separation of multi-channel eeg. Acta Biophys. Sinica 20, 77–82.

[B44] Zhang Y. D. Yang Z. J. Lu H. M. Zhou X. X. Phillips P. Liu Q. M. (2016). Facial emotion recognition based on biorthogonal wavelet entropy, fuzzy support vector machine, and stratified cross validation. IEEE Access 99, 1–1. 10.1109/ACCESS.2016.2628407

[B45] Zheng W. L. Zhu J. Y. Lu B. L. (2016). Identifying stable patterns over time for emotion recognition from eeg. IEEE Trans. Affect. Comput. 4, 8375–8385. 10.1109/TAFFC.2017.2712143

